# Does self-construal shape automatic social attention?

**DOI:** 10.1371/journal.pone.0246577

**Published:** 2021-02-10

**Authors:** Ronda F. Lo, Andy H. Ng, Adam S. Cohen, Joni Y. Sasaki

**Affiliations:** 1 Department of Psychology, York University, Toronto, Ontario, Canada; 2 Cardiff Business School, Cardiff University, Cardiff, Wales, United Kingdom; 3 Department of Psychology, University of Hawaiʻi at Mānoa, Honolulu, Hawaii, United States of America; Waseda University, JAPAN

## Abstract

We examined whether activating independent or interdependent self-construal modulates attention shifting in response to group gaze cues. European Canadians (Study 1) and East Asian Canadians (Study 2) primed with independence vs. interdependence completed a multi-gaze cueing task with a central face gazing left or right, flanked by multiple background faces that either matched or mismatched the direction of the foreground gaze. Results showed that European Canadians (Study 1) mostly ignored background gaze cues and were uninfluenced by the self-construal primes. However, East Asian Canadians (Study 2), who have cultural backgrounds relevant to both independence and interdependence, showed different attention patterns by prime: those primed with interdependence were more distracted by mismatched (vs. matched) background gaze cues, whereas there was no change for those primed with independence. These findings suggest activating an interdependent self-construal modulates social attention mechanisms to attend broadly, but only for those who may find these representations meaningful.

## Introduction

Humans easily infer what others want or feel by following their gaze [1, 2]. This ability—called *social attention*—is automatic [1], early developing [3], and often assumed to be resistant to external influence. Yet attention shifting to multiple gaze cues, even at the automatic level, varies in different cultures [4]. Although long-term exposure to different cultural contexts may impact the social attention system, it is unknown whether more proximal cultural information in the environment, such as cultural primes, can influence automatic processes in social attention. In the present research, we examine how activating different self-construals [5] can modulate automatic responses to gaze cues.

Central to literature on social attention is the gaze cueing paradigm: a directional gaze can facilitate reaction times to perceiving targets that eventually appear in the same (congruent) versus opposite (incongruent) direction [1, 6]. This “cueing effect” occurs despite cues being uninformative about where the target appears (i.e., when only 50% of cues are valid) and even when participants are explicitly informed that cues are nonpredictive [1], suggesting that gaze cues trigger attention shifting automatically. The cueing effect depends on stimulus onset asynchrony (SOA: interval between when cue and target appear), is triggered as early as 200 ms, typically persists until 600–700 ms, and dissipates or reverses towards 1,000 ms [1, 7], suggesting that the effect is automatic (under 700 ms).

The automatic cueing interval, however, can be penetrated by top-down knowledge (e.g., [2, 8]), including the cultural context. Cohen and colleagues [4] found that the cueing effect varied across cultures and SOA in a multi-gaze cueing task with background gazes looking in a different direction than the foreground gaze. While there were no cultural differences at the earlier SOA (200 ms), at the later automatic-interval SOA (600 ms), cultural differences emerged: East Asian participants, but not European Americans, experienced interference from incongruent background gazes, interrupting automatic attention shifting from the foreground gaze. This is consistent with other cultural psychological research that has demonstrated cognitive differences between North Americans and East Asians [9], and specifically, attention to background information, like faces’ emotional states [10, 11] when processing visual scenes with foreground and background faces (see [12, 13] for review). Cohen and colleagues’ [4] findings further suggest that cultural contexts may calibrate social attention mechanisms to attend narrowly (focused on a singular face) versus broadly (across multiple background faces).

Yet what remains unknown is the level of cultural information integrated in the social attention system. Findings from Cohen and colleagues [4] suggest that *distal* cultural factors can result in different settings for the social attention system due to repeated cultural inputs over time. However, can *proximal* factors that operate over much shorter timescales, such as cultural primes, influence the social attention system? Research suggests that proximal factors such as independent versus interdependent self-construals (i.e., representations of the self as separate from or connected to others; [5]) can elicit associated cognitive processes [14, 15] that are relevant to social attention. If thinking of the self as connected to others can broaden the mode of social attention to integrate multiple social cues, this implicates that the social attention system, and gaze cues in particular, may be less rigid than typically regarded and more susceptible to proximal information to navigate social environments that might necessitate attending to others’ mental states.

However, will priming self-construal affect social attention for any cultural group? There is evidence that both self-construals co-exist to varying degrees in all individuals and are activated by specific social domains [16], suggesting, on one hand, that priming different self-construals may activate mechanisms in the social attention system for any cultural group. On the other hand, these mechanisms may be well-practiced and difficult to change (i.e., uninfluenced by priming) if the social attention system has already been set from long-term exposure. It is currently unknown whether the social attention system will *prioritize* proximal cultural information that differs from pre-calibrated, culture-specific settings or whether the system will be rigid to proximal cultural information, maintaining pre-calibrated settings.

In Study 1, we examine whether activating independent versus interdependent self-construal can penetrate automatic mechanisms of social attention to attend more narrowly or broadly for European Canadians. In previous research, European Americans tended to exhibit a narrow mode of attention [4], consistent with independent self-construals, so whether European Americans will prioritize the integration of the interdependent self-construal prime over their pre-calibrated social attention settings is unknown.

Our predictions focus on conditions of *mismatched* cues, when foreground and background gaze directions do not match, in the automatic cueing interval (200 and 600 ms SOA). Given that prior research has successfully demonstrated that self-construal priming can shift visual attention [14, 15], we hypothesized that the social attention system would, too, be malleable to proximal cultural information in the environment. Specifically, we hypothesized that under mismatched conditions (i.e., background faces gaze in the opposite direction of the foreground face), European Canadians primed with independence should increase attention to the foreground gaze cue, which should result in significant cueing effects in the automatic cueing interval; interdependent primes should increase attention to both foreground and background, such that incongruent gazes in the background should interfere with processing foreground gazes that are congruent with the target, dampening cueing effects in the automatic cueing interval.

Given evidence that priming effects in gaze cueing may occur as early as 200 ms SOA [17, 18], but cueing effects for distal cultural factors in past research dissipated at 600 ms SOA when background gazes mismatched the foreground [4], we did not have expectations about whether the predicted priming effects should occur earlier (200 ms) or later (600 ms SOA) in the automatic cueing interval.

All data, analyses, and supplementary materials are available on Open Science Framework (https://osf.io/5pqdy/).

## Study 1

### Method

#### Participants and design

The research conducted in this article received ethics approval from the Office of Research Ethics at York University, Toronto, Canada. Ethics Certificate#: 2014–352. Written informed consent was obtained at the time of testing. The initial sample size of 138 European Canadian undergraduates was determined by the goal of collecting a minimum of 50 participants per prime group, adjusting for exclusions. Of these, 37 were excluded and removed prior to analyses: 31 did not meet cultural background criteria and 6 were unusable due to unforeseen circumstances (e.g., not following instructions). The final sample included 101 participants (*M*_age_ = 20.32 years, *SD*_age_ = 4.76; 71.29% female) who were of Western European descent and born in Canada, with at least one parent born in Canada. These criteria ensured a relatively uniform, monocultural cultural background, as the data collection location (Toronto) has a large proportion of recent European immigrants from countries in Southern, Central, and Eastern Europe known to be more interdependent cultures [19, 20]. We conducted a power analysis based on findings from Cohen and colleagues [4], with a 3-way interaction effect size of η_*p*_^2^ = 0.051. We made the conservative estimate that priming effects could be smaller than cultural group differences, so we used an η_*p*_^2^ = 0.031, or *f* = 0.17 as our estimation of a meaningful effect size to calculate power. Power analyses indicated that Study 1 has 99.2% power to detect *f* = 0.17 between Condition, SOA, and Prime, with an *N* = 101, number of groups = 2 (Prime: Independent vs. Interdependent), number of measurements = 6 (Condition: Matched, Mismatched × SOA: 200, 600, 1,000), correlation amongst repeated measures = 0.5 (conservative estimate), nonsphericity correction ε = 0.75 (conservative estimate).

This study utilized a 2 (Condition: Matched background and foreground gazes vs. Mismatched background and foreground gazes) × 3 (SOA: 200 ms vs. 600 ms vs. 1,000 ms) × 2 (Prime: Independent vs. Interdependent) mixed design, with repeated measures on the first two factors.

#### Apparatus and stimuli

The experiment was presented on a monitor 47.5cm (L) × 29.5 cm (W) with 1680 × 1050 pixel-resolution, positioned 60 cm away from participants. A 7-inch ICU Personal Convex Mirror was attached to the top center of the monitor and angled downwards, so that participants’ eye movements would be noticeable to the experimenter sitting behind the participant. This setup, used in previous social attention research requiring eye-movement monitoring [21], allowed the experimenter to check that participants did not make eye movements before target onsets.

**Fig 1:**
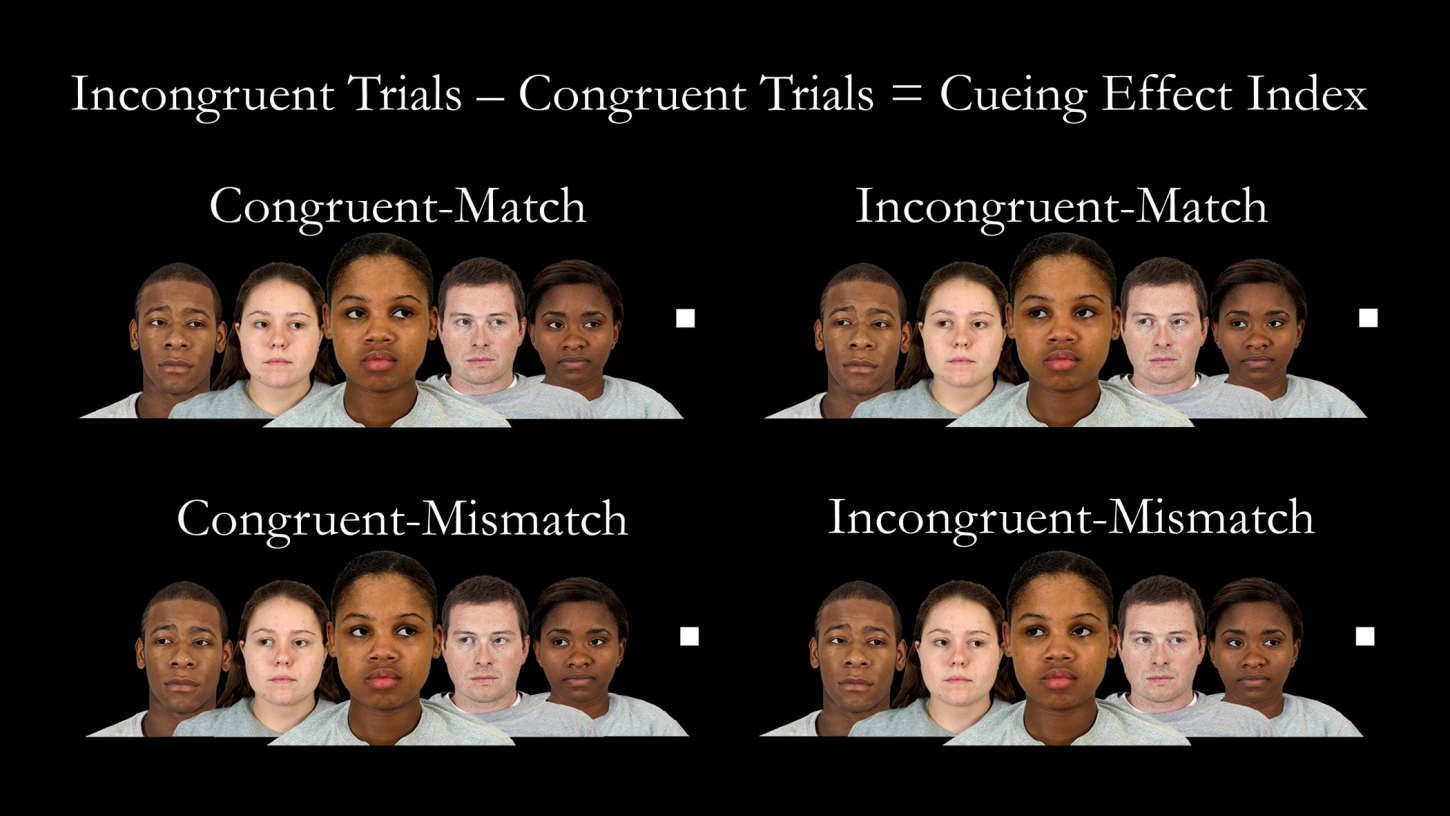
Types of trials in the multi-gaze cueing task. *Note*: The foreground gaze was either directed away from (incongruent, left column) or towards (congruent, right column) the target (white square), and either matched (top row) or mismatched (bottom row) background gaze cues. The cueing effect index was calculated by subtracting congruent from incongruent trial reaction times. Faces shown here are for illustrative purposes only (from the Chicago Face Database [22]) and do not reflect face stimuli from the multi-gaze cueing task. For actual faces used, see Cohen and colleagues’ paper [4].

The multi-gaze cueing task [4], run with E-Prime Software (2.0.8.90), presented a foreground face in the center of the screen flanked by two background faces on each side (four background faces total; Fig 1). Each of the five faces was randomly selected from a set of 12 faces varying in gender and ethnicity, and the target was a white square (stimuli measurements in Table 1).

**Table 1:** Stimuli measurements for multi-gaze cueing task.

		Measurements
Stimuli	Centimeters	Degrees of Visual Angle
*Background faces*		
Distance from center of screen to center of image		
Left- and rightmost face	10.05	9.57°
Second left- and rightmost face	5.65	5.39°
Image size		
Whole face (left to right edge of face)	4.90	4.68°
Eye region (leftmost part of left eye to rightmost part of right eye)	3.30	3.15°
Left eye (leftmost to rightmost part of left eye)	0.95	0.91°
*Foreground face*		
Distance from center of screen to center of image	0	0°
Image size		
Whole face (left to right edge of face)	6.50	6.20°
Eye region (leftmost part of left eye to rightmost part of right eye)	3.85	3.68°
Left eye (leftmost to rightmost part of left eye)	1.65	1.58°
*Target*		
Distance from center of screen to center of image	15.30	14.53° (left or right side)
Image size	1.90	1.81°

*Note*: Centimeters refer to size measured on the physical monitor. Degrees of visual angle (units to measure size of stimulus on the retina) were calculated with a distance of 60 cm between participant and monitor.

#### Materials

*Pronoun circling task*. Participants were randomly assigned to either the independent or interdependent prime from the Pronoun Circling Task [23]. To safeguard against priming effects weakening across trial blocks, four different versions of the task (the original plus 3 created versions) were used per prime. The participant completed a different version of the task before each trial block, with all versions priming the same self-construal across the four experimental blocks for each.

Four set orders for the versions were created via Latin square design, and each participant was assigned an order randomly without replacement.

*Additional measures*. The Self-Construal Scale [24] and Analysis-Holism Scale [25] were included for exploratory purposes (reported in supplementary materials), and a measure of social inference (adapted from [26]) was included but not analyzed for this study.

#### Procedure

There were 520 trials in total, with 240 test trials (40 trials in each match × SOA condition), 240 filler trials, and 40 catch trials. Trials were evenly distributed across 4 blocks (130 trials per block). On test trials, foreground and background faces gazed equally and randomly to the left and right, and the target appeared equally and randomly to the left and right of faces. SOA condition was selected randomly without replacement. The central cue was congruent with the target on half the trials and incongruent on the other half. Congruency was pseudorandom. Filler trials were like test trials except foreground and background faces gazed forward, rather than left or right. These were included to create stimulus diversity, so that participants could not easily predict gaze direction on each trial. On catch trials, no target appeared to ensure participants were paying attention to the target and not anticipating a left or right response from gaze cues.

Each trial had three phases. In the closed phase, all faces appeared with eyes closed for 500 ms, and the fixation dot was positioned on the foreground face between the eyes, alongside background faces. In the cue phase, foreground and background faces gazed either left or right. All background faces gazed in the same direction and were just as likely to be congruent as incongruent with the foreground face’s gaze direction. In the target phase, cues remained on-screen while the target appeared to the left or right of the faces after 200, 600, or 1,000 ms from cue onset. Trials ended if participants responded with a spacebar press or if there was no response after 2,000 ms from target onset.

Participants’ reaction times (RTs) were recorded from target onset to the spacebar response. We processed RT data by removing anticipation trials (RTs less than 100 ms), expiration trials (trials without a response after 2,000 ms), and outliers (RTs beyond two standard deviations from the grand mean per participant at each level of SOA) (Table 2). We then computed the dependent variable (i.e., cueing effect index) by subtracting RTs on congruent trials (i.e., when foreground gaze predicted target location) from RTs on incongruent trials (i.e., when foreground gaze did not predict target location) for each SOA and Condition (i.e., whether foreground and background gaze direction matched or not) (Tables 3 and 4). This resulted in 6 cue effect indices per participant (Condition × SOA). A positive index indicates a stronger cueing effect from the foreground gaze, an index not different from zero indicates no cueing effect, and a negative index indicates a stronger reverse cueing effect.

**Table 2:** Data cleaning analysis for European Canadians.

	Criteria	Percentage of All Trials (%)
Outlier Trials	Reaction time to detect target was 2 SD above or below the grand mean across all trials per participant at each SOA	4.34%
Anticipation Trials	Reaction time to detect target was less than 100 ms	0.26%
Expiration Trials	Reaction time was greater 2,000 ms, indicating no response was made to locate target	0.36%

**Table 3:** Raw reaction times for European Canadians.

	Matched Condition				Mismatched Condition			
	Congruent–Congruent		Incongruent–Incongruent		Congruent–Incongruent		Incongruent–Congruent	
	*M*	*SE*	*M*	*SE*	*M*	*SE*	*M*	*SE*
Independent								
200 ms	352.10	7.68	360.95	7.59	354.83	8.25	358.45	7.06
600 ms	322.31	6.30	333.91	7.75	323.68	6.61	326.32	6.55
1,000 ms	334.69	6.53	329.08	7.27	323.54	6.38	330.32	7.65
Interdependent								
200 ms	363.98	8.48	371.94	7.89	365.43	8.28	371.67	8.37
600 ms	337.80	7.43	345.56	7.64	337.14	6.97	339.03	7.73
1,000 ms	334.32	6.81	336.56	6.73	333.33	6.12	336.91	7.06

*Note*: Means (*M*) and standard errors (*SE*) of reaction times by Condition (Matched vs. Mismatched), Cue direction (Congruent vs. Incongruent foreground–background), Prime (Independent vs. Interdependent), and Stimulus Onset Asynchrony (SOA; 200 vs. 600 vs. 1,000 ms) for European Canadians.

**Table 4:** Cueing effect indices for European Canadians.

	Matched Condition			Mismatched Condition		
	*M*	*SE*	95% CI	*M*	*SE*	95% CI
Independent						
200ms	8.85	2.38	[4.08, 13.62]	3.62	3.51	[-3.42, 10.66]
600 ms	11.60	3.52	[4.54, 18.66]	2.65	2.36	[-2.10, 7.39]
1,000 ms	4.40	2.77	[-1.16, 9.96]	6.78	2.77	[1.22, 12.35]
Interdependent						
200 ms	7.95	3.20	[1.52, 14.39]	6.24	2.62	[0.96, 11.51]
600 ms	7.76	3.06	[1.61, 13.91]	1.90	2.88	[-3.90, 7.69]
1,000 ms	2.23	2.24	[-2.28, 6.74]	3.58	3.05	[-2.55, 9.72]

*Note*: Means (*M*), standard errors (*SE*), and 95% mean confidence intervals of cueing effects by subtracting congruent foreground trials from incongruent foreground trials by Condition (Matched vs. Mismatched), Prime (Independent vs. Interdependent), and Stimulus Onset Asynchrony (SOA; 200 vs. 600 vs. 1,000 ms) for European Canadians.

The experimenter instructed the participant to look at the central fixation point, and to respond with a spacebar press with their dominant hand’s index finger when they see the target appear (experimenter instructions are available in an online supplementary file). They did not need to respond if no target appeared. Participants were also told the purpose of the mirror and to ignore it. The experimenter sat approximately 90 cm behind participants to track their gazing.

### Results and discussion

**Fig 2:**
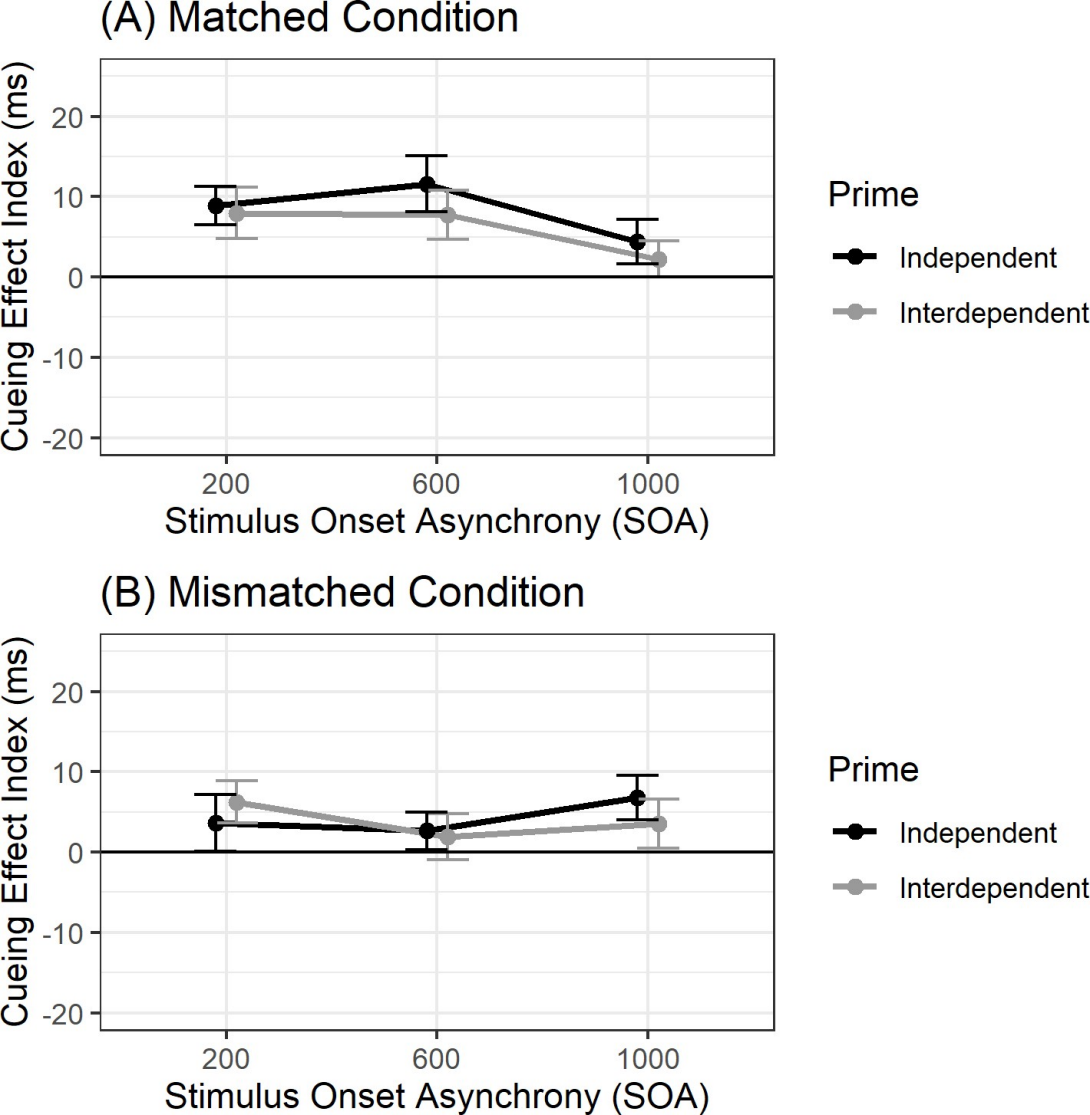
Cueing effects for European Canadians. *Note*: Cueing Effect Index as a function of SOA and Prime for (A) matched and (B) mismatched conditions. Error bars represent standard error of the mean. Participants are European Canadians.

A mixed 2 × 3 × 2 ANOVA revealed a marginal main effect of Condition: the cueing effect was stronger under matched than mismatched conditions (Tables 3 and 4), *F*(1, 99) = 3.73, *p* = .056, η_*p*_^2^ = .04. There was a marginal interaction between Condition and SOA, *F*(2, 198) = 2.83, *p* = .061, η_*p*_^2^ = .03. There was no main effect of Prime, *F*(1, 99) = 0.52, *p* = .472, η_*p*_^2^ = .005, and there were no two- or three-way interactions of Prime with Condition and SOA, *ps >* .*55*, η_*p*_^2^s < .004, suggesting the primes did not shift the social attention system into narrower or wider modes of attention (Fig 2).

To investigate the nature of the Condition × SOA interaction, we conducted one-way ANOVAs of Condition on cueing effects by each SOA separately. Under 200 ms SOA, there was no effect of Condition, *F*(1, 100) = 1.70, *p* = .195, η_*p*_^2^ = .02, suggesting cueing effects did not differ whether foreground and background gazes matched or mismatched. One sample *t*-tests against zero show that cueing effects are significantly above zero under both matched, *t*(100) = 4.28, *p* < .001, *d* = 0.43, and mismatched conditions, *t*(100) = 2.22, *p* = .029, *d* = 0.22.

Under 600 ms SOA, there was a significant effect of Condition, *F*(1, 100) = 6.24, *p* = .014, η_*p*_^2^ = .06, where cueing effects under matched conditions were significantly greater than mismatched conditions (Table 4). One sample *t*-tests showed significant cueing effects under matched conditions, *t*(100) = 4.16, *p* < .001, *d* = 0.41, and no cueing effects under mismatched conditions, *t*(100) = 1.24, *p* = .219, *d* = 0.12. The lack of cueing effect under mismatched conditions at 600 ms SOA was unexpected and looks similar to East Asians under the same conditions in prior research [4], suggesting European Canadians, under mismatched conditions, may have broadened their scope of attention to incorporate both foreground and background gaze cues. This may be a result of our European Canadians being more broadly interdependent—and similar to East Asians [4]—than initially hypothesized. Research with other Europeans from the local context (i.e., Toronto) has suggested that non-Western European culture is highly salient, and that these European-descent individuals retain languages, cultural attitudes, and beliefs, from their heritage [19].

There was no significant effect of Condition under 1,000 ms SOA, *F*(1, 100) = 0.58, *p* = .450, η_*p*_^2^ = .006, suggesting cueing effects did not differ whether foreground and background gazes matched or mismatched. One sample *t*-tests against zero show that cueing effects were not significantly above zero for matched, *t*(100) = 1.87, *p* = .064, *d* = 0.19, but were above zero for mismatched conditions, *t*(100) = 2.55, *p* = .012, *d* = 0.25. The cueing effect re-occuring at 1,000 ms SOA was unexpected, but as it falls outside of the automatic cueing interval, it does not provide a meaningful understanding of whether the primes penetrated automatic mechanisms for social attention, and furthermore, there was no difference in cueing effects between primes.

In addition to our Frequentist (referred to as NHST: Null Hypothesis Significance Testing) analyses, we also conducted Bayesian analyses. A Bayesian ANOVA with a default prior, r = 0.5 (fixed effects), showed the data support a null model (including all lower order effects) over the alternative 3-way model (15.6:1 odds in favor of the null relative to the alternative, or BF_01_ = 15.6), which is consistent with our NHST analyses that the primes did not interact with match condition or SOA.

Overall, European Canadians did not shift their patterns of social attention in response to the manipulation, suggesting their social attention system was not malleable to self-construal primes. This may suggest at first glance that the social attention system, broadly, is relatively inflexible to proximal factors. However, this seems somewhat unlikely given previous research demonstrating priming effects on the social attention system [17, 18]. Instead, it may be the case that the extent to which priming can influence the social attention system should depend on familiarity of the prime [27]. In the context of the current study, in order for the social attention system to shift narrowly and broadly in response to independent and interdependent primes, respectively, the social attention system may already need experience with switching between narrow and broad modes of attention in response to environmental cues of independence and interdependence. A crucial sample to test this would be biculturals, who engage with both a mainstream and heritage culture [28]. Given that biculturals often experience switching between cultures, they may also be well-practiced in switching between different modes of social attention, so they may be more responsive to the primes. Thus, in Study 2, we test the impact of priming self-construal on the social attention system for biculturals.

## Study 2

We pre-registered Study 2 to test our hypotheses with East Asian Canadian biculturals using identical analyses from Study 1 (https://osf.io/yzvmt/). Given that East Asian Canadians may be more well-practiced than European Canadians at shifting between self-construals as demanded by their different cultural contexts, we hypothesized that East Asian Canadians should respond to the prime such that those primed with independence would exhibit a narrow mode of attention with cueing effects appearing across both matched and mismatched conditions. East Asian Canadians primed with interdependence, however, should exhibit a broader mode of attention with dampened cueing effects under mismatched conditions.

### Method

#### Participants and design

The research conducted in this article received ethics approval from the Office of Research Ethics at York University, Toronto, Canada. Ethics Certificate#: 2014–352. Written informed consent was obtained at the time of testing. The final sample included 102 East Asian Canadian undergraduates (*M*_age_ = 19.70 years, *SD*_age_ = 2.62; 55.88% female) who had not lived in East Asia for more than 16 years, with both parents born in East Asia. These criteria ensured participants were sufficiently exposed to both East Asian and Canadian culture. These also mirror the requirements Cohen and colleagues [4] had for their East Asian monocultural sample who lived in East Asia for at least 16 years. Out of the final sample, 37 were not born in Canada (age of arrival: *M* = 7.54 years, *SD* = 4.50). The initial sample size was 164 participants, as we aimed to meet the number of Study 1 participants (*N* = 101), while oversampling to account for East Asian Canadians who would not meet our inclusion criteria. Sixty-two participants were excluded from the data: 47 participants were removed for not meeting the cultural background inclusion criteria (e.g., living in East Asia for more than 16 years, one parent born in Canada, etc.), and 15 whose data were unusable due to external circumstance (e.g., participant not following instructions). Participants were removed prior to data cleaning (Table 5). Power analysis indicated that Study 2 has 99.3% power to detect a small interaction (*f* = 0.17, or η_*p*_^2^ = 0.03) between Condition, SOA, and Prime conditions using the same parameters and justifications as Study 1, except for the sample size (*N* = 102).

**Table 5:** Data cleaning analysis for East Asian Canadians.

	Criteria	Percentage of All Trials (%)
Outlier Trials	Reaction time to detect target was 2 SD above or below the grand mean across all trials per participant at each SOA	1.66%
Anticipation Trials	Reaction time to detect target was less than 100 ms	0.12%
Expiration Trials	Reaction time was greater 2,000 ms, indicating no response was made to locate target	0.20%

*Note*: The design was the same as in Study 1: a 2 (Condition: Matched vs. Mismatched) × 3 (SOA: 200 ms vs. 600 ms vs. 1,000 ms) × 2 (Prime: Independent vs. Interdependent) mixed design, with repeated measures on the first two factors.

**Apparatus, stimuli, materials and procedure.** These were identical to Study 1.

### Results and discussion

**Fig 3:**
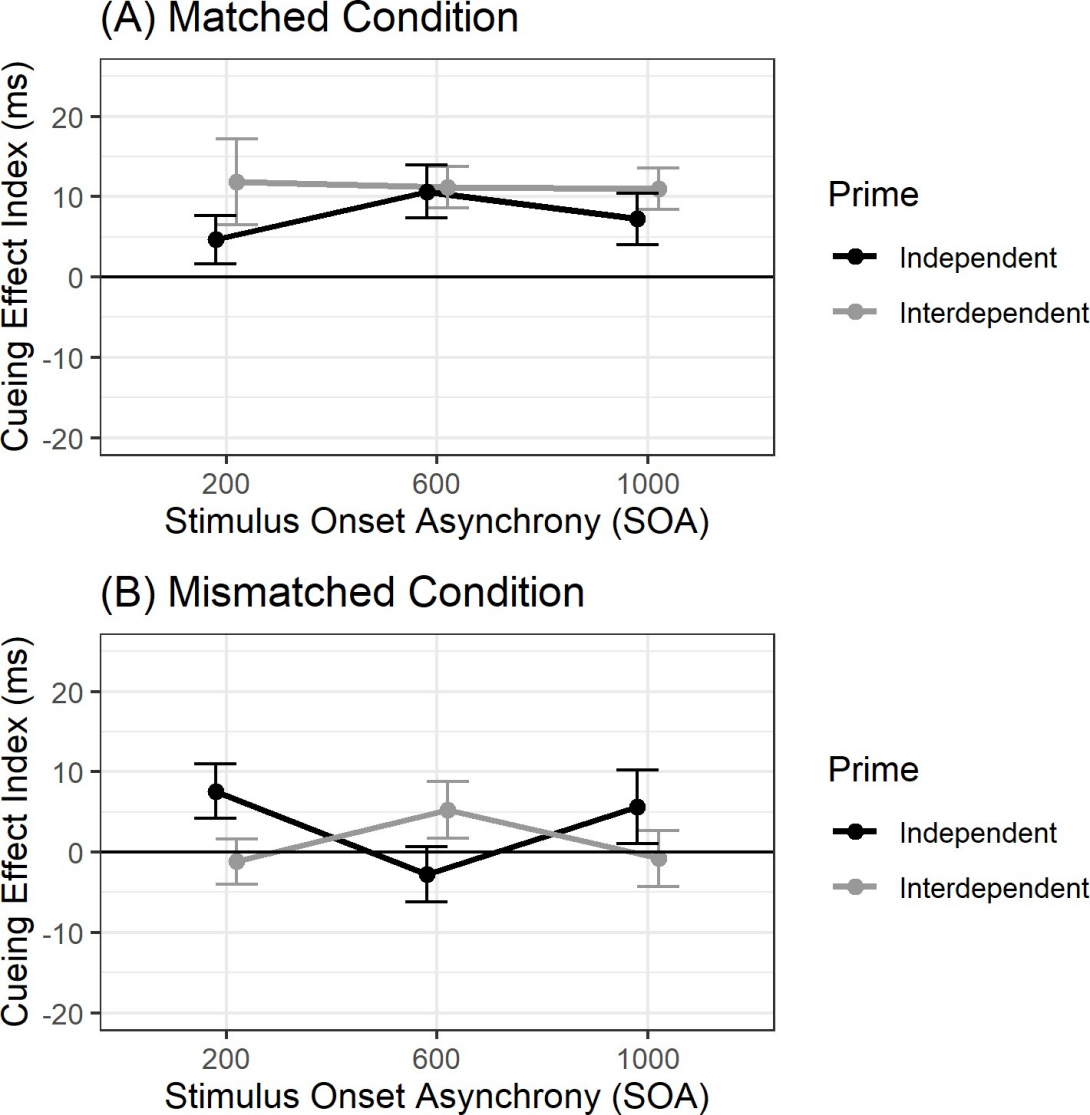
Cueing effects for East Asian Canadians. *Note*: Cueing Effect Index as a function of SOA and prime for (A) matched and (B) mismatched conditions. Error bars represent standard error of the mean. Participants are East Asian Canadians.

A mixed 2 × 3 × 2 ANOVA revealed a main effect of Condition: the cueing effect was stronger under matched than mismatched conditions (Tables 6 and 7), *F*(1, 100) = 20.02, *p* < .001, η_*p*_^2^ = .17. There was a marginal interaction between Prime and Condition, *F*(1, 100) = 3.84, *p* = .053, η_*p*_^2^ = .04. This was qualified by a marginal 3-way interaction between Prime, Condition, and SOA, *F*(2, 200) = 2.94, *p* = .055, η_*p*_^2^ = .03 (Fig 3). The effect size of this 3-way interaction was accurately estimated from our power analyses where we conservatively estimated a potential effect size of η_*p*_^2^ = .031. Cohen and colleagues [4] estimated η_*p*_^2^ = .051 from their 3-way Culture × Condition × SOA interaction, which can be found in our 3-way Prime × Condition × SOA interaction effect size confidence interval, 95% CI [0, 0.08], suggesting comparable effect size estimations between the two studies. Nevertheless, we acknowledge that our findings are not significant by NHST standards.

**Table 6:** Raw reaction times for East Asian Canadians.

	Matched Condition				Mismatched Condition			
		[Congruent–Congruent]		[Incongruent–Incongruent]		[Congruent–Incongruent]		[Incongruent–Congruent]	
	*M*	*SE*	*M*	*SE*	*M*	*SE*	*M*	*SE*
Independent								
200 ms	389.08	19.24	393.72	19.06	386.13	19.15	393.73	19.83
600 ms	346.54	14.66	357.19	16.58	354.34	16.82	351.61	15.15
1,000 ms	346.48	13.89	353.72	14.59	345.07	12.75	350.72	15.51
Interdependent								
200 ms	369.03	11.35	380.87	13.19	379.48	12.50	378.31	12.69
600 ms	339.28	10.75	350.47	10.65	342.81	9.91	348.09	11.55
1,000 ms	337.76	9.56	348.78	10.37	341.29	10.18	340.51	10.78

*Note*: Means (*M*) and standard errors (*SE*) of reaction times by Condition (Matched vs. Mismatched), Cue direction (Congruent vs. Incongruent: foreground–background), Prime (Independent vs. Interdependent), and Stimulus Onset Asynchrony (SOA; 200 vs. 600 vs. 1,000 ms) for East Asian Canadians.

**Table 7:** Cueing effect indices for East Asian Canadians.

	Matched Condition			Mismatched Condition		
	M	SE	95% CI	M	SE	95% CI
Independent						
200 ms	4.64	2.97	[-1.32, 10.60]	7.59	3.35	[0.89, 14.30]
600 ms	10.64	3.30	[4.03, 17.25]	-2.73	3.44	[-9.61, 4.15]
1,000 ms	7.24	3.18	[0.87, 13.60]	5.65	4.55	[-3.46, 14.75]
Interdependent						
200 ms	11.85	5.30	[1.16, 22.54]	-1.17	2.82	[-6.87, 4.52]
600 ms	11.19	2.60	[5.94, 16.43]	5.27	3.52	[-1.82, 12.37]
1,000 ms	11.02	2.57	[5.83, 16.20]	-0.78	3.50	[-7.84, 6.28]

*Note*: Means (*M*), standard errors (*SE*), and 95% mean confidence intervals of cueing effects by subtracting congruent foreground trials from incongruent foreground trials by Condition (Matched vs. Mismatched), Prime (independent vs. interdependent), and Stimulus Onset Asynchrony (SOA; 200 vs. 600 vs. 1,000 ms) for European Canadians.

To examine the nature of the 3-way interaction, data were analyzed separately by SOA. Under 200 ms SOA, a 2 × 2 (Prime × Condition) mixed ANOVA revealed an interaction between Prime and Condition, *F*(1, 100) = 5.91, *p* = .017, η_*p*_^2^ = .06. The 95% confidence interval of the Prime × Condition interaction, 95% CI [0.001, 0.16], also includes the parallel 2-way interaction effect size found in Cohen and colleagues [4] (η_*p*_^2^ = .058). The interaction emerged because of a difference between match and mismatch conditions for interdependent primes but not for independent primes. Specifically, for interdependent primes, the cueing effect was larger when the gaze cues matched (*M* = 11.84 ms, *SD* = 35.58 ms) than when they mismatched (*M* = -1.17 ms, *SD* = 18.95 ms), *t*(100) = 2.65, *p*_*tukey*_ = .046, *d* = 0.26. One sample *t*-tests against zero demonstrated significant cueing effects under matched conditions, *t*(44) = 2.23, *p* = .031, *d* = 0.33, but not under mismatched conditions, *t*(44) = -0.42, *p* = .680, *d* = 0.06. For independent primes, the cueing effect did not differ between matched (*M* = 4.64 ms, *SD* = 22.45 ms) and mismatched conditions (*M* = 7.59 ms, *SD* = 25.26 ms), *t*(100) = -0.68, *p*_*tukey*_ = .91, *d* = 0.06. (Table 7). One sample *t*-tests against zero were significant for the mismatched condition, *t*(56) = 2.27, *p* = .03, *d* = 0.30, but not for the matched condition, *t*(56) = 1.56, *p* = .12, *d* = 0.21. This suggests that those primed with interdependence, but not independence, integrated background gaze cues, which interfered with processing foreground gaze cues at an early stage of the automatic cueing interval.

Under 600 ms SOA, a 2 × 2 (Prime × Condition) mixed ANOVA revealed a main effect of Condition, *F*(1, 100) = 7.42, *p* = .008, η_*p*_^2^ = .07, where matched conditions produced greater cueing effects than mismatched conditions. The predicted Prime × Condition interaction was not significant, *F*(1, 100) = 1.11, *p* = .295, η_*p*_^2^ = .01. Collapsing across prime groups, those in the matched condition produced cueing effects above zero, *t*(101) = 5.03, *p* < .001, *d* = 0.50, but those in the mismatched condition did not, *t*(101) = 0.32, *p* = .748, *d* = 0.03.

For those primed with independence under mismatched conditions, experiencing a cueing effect at 200 ms SOA, but not at 600 ms SOA suggests the background gazes interfered with foreground gaze cueing at a later (i.e., 600 ms) SOA, replicating previous effects found for unprimed East Asians [4]. Those primed with interdependence under mismatched conditions, however, experienced no cueing effect across both 200 and 600 ms SOA, suggesting that manipulating self-construal can impact social attention mechanisms even earlier than distal cultural factors previously shown to affect gaze cueing at 600 ms SOA [4]. Importantly, the current study’s effect sizes are comparable to Cohen and colleagues’ [4] previous findings, suggesting that self-construal may be one aspect of the cultural environment that can program the social attention system.

Under 1,000 ms SOA, a 2 × 2 (Prime × Condition) mixed ANOVA revealed a main effect of Condition, *F*(1, 100) = 4.64, *p* = .034, η_*p*_^2^ = .04, where matched conditions produced greater cueing effects than mismatched conditions. The Prime × Condition interaction was not significant, *F*(1, 100) = 2.70, *p* = .104, η_*p*_^2^ = .03. Again, collapsing across prime groups, those in the matched condition produced cueing effects above zero, *t*(101) = 4.23, *p* < .001, *d* = 0.42, but those in the mismatched condition did not, *t*(101) = 0.94, *p* = .35, *d* = 0.09. The persistent cueing effect at 1,000 ms SOA under mismatched conditions was unexpected, but again, cueing effects outside the automatic cueing interval are uninterpretable in the context of the current research question. A 1,000 ms condition was included in part to conform to prior studies which typically include a long SOA and in part to prevent participants from predicting when the target would appear, forcing them to attend to where it appeared. If participants know when a target will appear, they can press the response key after the known delay without needing to attend to where it appears, undermining the logic of the spatial gaze cueing paradigm.

The Bayesian analysis allows clearer interpretation of the direction of evidence towards the null or alternative: A Bayesian ANOVA (all analyses described used a default prior, r = 0.5, fixed effects) showed the data barely support the alternative 3-way model over the null model (including all lower order effects), with 1.5:1 odds in favor of the alternative (BF_10_ = 1.5). The support is not strong (Bayes factors are considered “substantial” when they are at least greater than 3; [29]). At the 200 ms SOA, the data support models containing the Condition × Prime interaction over equivalent models without the effect, BF_incl_ = 3.25, but at the 600 ms SOA, the data most strongly favor models with only the Condition main effect, BF_incl_ = 19.3, consistent with NHST analyses.

For the interdependent prime group, Bayesian *t*-tests (all tests described used a default Cauchy prior, width = 1/√2) found evidence for the null (cueing effect not different from zero) over the alternative hypothesis (cueing effect greater than zero) at the 200 ms SOA, BF_01_ = 5.70, and weak evidence for the null at the 600 ms SOA, BF_01_ = 2.19. For the independent prime group, the evidence favored the null over the alternative at the 600 ms SOA, BF_01_ = 5.12 but not at the 200 ms SOA, BF_01_ = 0.65.

In additional analyses, we examined whether including birth status (i.e., being born in Canada or not) as a covariate resulted in differences in this 3-way interaction, given that some participants were born in Canada (*n* = 65), and some were born elsewhere but arrived in Canada at a very early age (*n* = 37). Birth status had a significant effect, *F*(1, 99) = 5.31, *p* = .023, η_*p*_^2^ = .05. Those who were born in Canada had smaller cueing effects, *M* = 3.65, *SE* = 1.16, 95% CI [1.37, 5.39], than those who were not born in Canada, *M* = 9.64, *SE* = 1.95, 95% CI [5.80, 13.50]. Larger cueing effects for non-Canadian born participants suggests they were faster to disengage from congruent trials, compared to Canadian-born participants. After accounting for birth status, the 3-way interaction between Prime, SOA, and Condition becomes conventionally significant, and the original effect size is identical, *F*(2, 198) = 3.14, *p* = .045, η_*p*_^2^ = .03. These covariate results suggest that birth status did not substantially influence the presented results.

## General discussion

This research demonstrates that proximally activated self-construals can influence mechanisms of social attention, but crucially, this effect existed only among biculturals (Study 2), who should be sensitive to both independent and interdependent self-construals, and not monoculturals (Study 1). Indeed, we provided some initial evidence that self-construal primes may only shift the social attention system for cultural groups such as East Asian biculturals that likely have experience shifting their attention to attend more narrowly or more broadly in response to culturally relevant cues in their environment.

When primed with interdependence, East Asian Canadians were influenced by mismatched gaze cues. Interdependence broadened their scope of attention, leading them to attend more to background gaze cues and interfering with attention shifting in response to the foreground gaze cue. We initially considered that priming effects could happen either at 200 ms SOA, because priming can immediately activate related concepts [17, 18], or at 600 ms SOA, because research shows distal cultural influences penetrate the social attention system after a minimum amount of time has passed [4]. In the present research, interdependent priming reduced the cueing effect across both 200 *and* 600 ms SOA, suggesting proximal cultural factors, such as primed self-construal, can have a quicker and longer lasting influence on the social attention system than even distal cultural factors [4].

When primed with independence, East Asian Canadians unexpectedly were still influenced by mismatched gaze cues and showed no cueing effect at the later automatic cueing interval, 600 ms SOA, while the cueing effect did appear at the earlier, 200 ms SOA, similar to previous findings with monocultural East Asians that were not primed with self-construal [4]. Although in the current study we expected that East Asian Canadians primed with independence would show the cueing effect at both 200 ms and 600 ms SOA, it is possible that East Asian Canadians have a primarily interdependent self-construal, and the proximal independence prime was not enough to switch the social attention system into a narrow scope of attention in the later automatic cueing interval.

Yet, importantly, automatic cueing patterns differed for East Asian Canadians depending on whether they were primed with interdependence or independence. These results suggest that highlighting the self as separate or connected to others can change attentional strategies at the automatic level. Even for the same group of biculturals—in this case, East Asian Canadians—it seems that their social attention system may operate differently at an automatic level depending on whether they think of themselves as independent or interdependent in the moment. Previous research has also demonstrated that priming proximal information can result in different attention strategies [14, 15, 30]. Yet the current research demonstrates that even at the automatic level, self-construal is an important aspect of the cultural environment that can program the social attention system. More broadly, the visual attention system may allow itself to be continuously shaped throughout adulthood by proximal environmental influences to adjust across varying social environments. Biculturals may develop the capacity for different attention strategies and readily integrate social orientation information to shift attentional settings to suit their current social environments.

The current research demonstrates the necessity of examining multiple cultural groups to test how predictions generalize to other populations. If we had only conducted this study with European Canadians (Study 1), we would have incorrectly concluded that the social attention system could not be modulated by proximal cultural information in the environment and that the calibration of the social attention system remains relatively rigid from early development. Yet although European Canadians were indeed uninfluenced by self-construal primes in Study 1, Study 2 demonstrated that East Asian Canadians were influenced by priming and diverged from the typical automatic cueing effect: when foreground and background gazes mismatched, East Asian Canadians seemed to find these mixed signals distracting, particularly after the interdependent prime.

This divergent effect of self-construal primes on social attention shifting for monoculturals vs. biculturals has implications for understanding how proximal cultural information is integrated into not only the social attention system, but also the visual attention system more broadly. Our findings suggest that proximally activated information must be easily accessible and well-practiced in order to impact bottom-up, attentional processes. Similar findings have also been demonstrated in previous research with self-construal priming and biculturals [26, 31, 32].

At first glance, these findings of self-construal priming on biculturals collectively seem to contradict research suggesting the co-existence of different self-construals in any cultural group [16] and self-construal priming effects on visual attention in multiple cultural groups [14, 15]. Whether or not self-construal priming effects can shape visual attention, however, may depend on task conditions. Hong and Chiu suggested that the use of culture-specific strategies may be more likely to occur under conditions of high cognitive load or when rapid decision making is required, which may encourage the use of culturally engrained strategies [33]. This might suggest that with the multi-gaze cueing task, where rapid decision making is required, European Canadian monoculturals defaulted to culturally engrained strategies, which was to maintain a narrow mode of attention. In contrast, East Asian Canadian biculturals may not be deeply attached to a culturally engrained strategy as strongly as monoculturals; their experience deploying both narrow and broader modes of attention might result in an attention system that is more malleable to proximal influences and can flexibly shift between different modes of attention.

Another potential reason why priming failed to shift European Canadians’ modes of attention in the current study may be that the multi-gaze cueing task demands rapid responses to complex social stimuli, as opposed to non-social stimuli (e.g., natural scenes, dots) used by past studies to test the effects of self-construal priming on attention [14, 15]. Prior experience shifting between modes of attention may not be as important for non-social stimuli as it is for social stimuli because the latter is associated with social norms of appropriate behavior. In the current study’s context, European Canadians may not have as much experience reflexively attending to the social cues of the group, resulting in rigid orienting to the individual.

The current findings also have implications for elucidating psychological mechanisms that underlie known cultural differences in visual attention. Social orientation (towards independent or interdependent thinking) is largely considered the explanatory mechanism that underlies cultural variation in analytical or holistic thinking [34]. Results from Study 2 suggest that when East Asian Canadians are primed with an interdependent self-construal, they tend to deploy a broader mode of attention to integrate background gaze cues, which supports the proposed link between interdependence and holistic thinking. Our findings provide initial evidence that shifts in social orientation can cause variation in automatic social attention strategies. Furthermore, our findings extend cultural psychological research beyond group comparison research by explicitly manipulating proposed proximal mechanisms that underlie cultural variation in cognition (see [35] for more on proximal vs. distal influences that underlie cultural variation in cognition).

We found significant cueing effects at 1,000 ms SOA under some matched and mismatched conditions. In a “single face” gaze-cueing task, the cueing effect typically disappears or reverses (gaze cues facilitate detecting targets that appear in the opposite direction) by 1,000 ms SOA [36, 37] (this is not always the case, however; see [38]). In a task with complex social stimuli in the background, disappearing or reversed cueing effects may not be as typical at 1,000 ms SOA, so using prior research from other tasks to understand what happens at long SOAs in the multi-gaze cueing task may be misleading. Considering that 1,000 ms SOA is when voluntary attention shifting can occur, the mixed results at 1,000 ms SOA should not impact the findings found within the automatic cueing interval.

We did not include a control condition in which participants received no self-construal prime. A control condition would be useful for interpreting certain priming effects across the two studies, illuminating whether the independent prime is influencing attention shifting from baseline, the interdependent prime is influencing attention shifting from baseline, or both. However, this also raises the question of what exactly a “baseline” should be for biculturals. The way in which unprimed biculturals would react to this task is unclear because biculturals have multiple ways of negotiating their cultural backgrounds and associated values [39]. These different bicultural negotiation strategies may then result in different “baseline” patterns of attention shifting. Future research using this task on unprimed biculturals, perhaps together with specific biculturalism measures, would help document how acculturation shapes automatic social attention processes.

Overall, these findings suggest that proximal cultural factors can shape social attention mechanisms to attune to culturally important information in a complex social environment, and that priming interdependent self-construal can further activate a broader scope of attention in the social attention system. These self-construals may also have different downstream consequences for those who attach significance to them. It is imperative, then that social attention research includes people from different societies since distal sociocultural experiences not only calibrate the social attention system itself, but also interface with proximal cultural factors to shape social attention.
